# Accelerating breast MRI acquisition with generative AI models

**DOI:** 10.1007/s00330-024-10853-x

**Published:** 2024-08-01

**Authors:** Augustine Okolie, Timm Dirrichs, Luisa Charlotte Huck, Sven Nebelung, Soroosh Tayebi Arasteh, Teresa Nolte, Tianyu Han, Christiane Katharina Kuhl, Daniel Truhn

**Affiliations:** Department of Radiology, University Hospital RWTH Aachen, Aachen, Germany

**Keywords:** Score-based models, Breast MRI reconstruction, Acceleration factors, Image quality

## Abstract

**Objectives:**

To investigate the use of the score-based diffusion model to accelerate breast MRI reconstruction.

**Materials and methods:**

We trained a score-based model on 9549 MRI examinations of the female breast and employed it to reconstruct undersampled MRI images with undersampling factors of 2, 5, and 20. Images were evaluated by two experienced radiologists who rated the images based on their overall quality and diagnostic value on an independent test set of 100 additional MRI examinations.

**Results:**

The score-based model produces MRI images of high quality and diagnostic value. Both T1- and T2-weighted MRI images could be reconstructed to a high degree of accuracy. Two radiologists rated the images as almost indistinguishable from the original images (rating 4 or 5 on a scale of 5) in 100% (radiologist 1) and 99% (radiologist 2) of cases when the acceleration factor was 2. This fraction dropped to 88% and 70% for an acceleration factor of 5 and to 5% and 21% with an extreme acceleration factor of 20.

**Conclusion:**

Score-based models can reconstruct MRI images at high fidelity, even at comparatively high acceleration factors, but further work on a larger scale of images is needed to ensure that diagnostic quality holds.

**Clinical relevance statement:**

The number of MRI examinations of the breast is expected to rise with MRI screening recommended for women with dense breasts. Accelerated image acquisition methods can help in making this examination more accessible.

**Key Points:**

- *Accelerating breast MRI reconstruction remains a significant challenge in clinical settings*.
- *Score-based diffusion models can achieve near-perfect reconstruction for moderate undersampling factors*.
- *Faster breast MRI scans with maintained image quality could revolutionize clinic workflows and patient experience*.

## Introduction

Breast MRI, with its superior soft tissue contrast and ability to visualize angiogenesis through contrast-enhanced techniques, offers distinct advantages over other modalities like ultrasound (US) and mammography for breast cancer detection [1]. Recently, the European Society for Breast Imaging (EUSOBI) published a consensus statement advocating routine screening for patients with dense breasts [2]. However, the wide-scale application of breast MRI is impeded by the high costs associated with MRI scanners and the required acquisition times. Consequently, there is a growing demand for accelerated image acquisition methods that can reduce examination time and thus increase the availability of breast MRI. A prevalent approach to accelerate MRI acquisition involves undersampling the k-space data and reconstructing the image data from this undersampled k-space data utilizing sensitivity encoding (SENSE) [3] or compressed sensing (CS) [4]. The advent of generative machine learning models [5] has extended these possibilities. Generative models can learn the distinguishing characteristics of typical images and can subsequently be used to synthesize new images that retain all the properties of real images [6]. This technology has shown promising applications in radiology, for example by reducing the need for contrast agents [7], and presents potential avenues for the acceleration of MRI acquisition [8, 9].

Among these generative models, score-based models offer a compelling approach [10–12]. These models operate within the framework of unsupervised learning. By integrating the generative model’s knowledge of what a typical MRI image would look like, ambiguities arising from missing k-space data can be resolved. This enables the reconstruction process to be completed even when some fractions of the k-space data are missing [13].

MRI reconstruction is an important technique in medical imaging. Traditional MRI reconstruction techniques can be time-consuming and computationally expensive, which can limit their practical use in clinical applications. However, recent advances in machine learning have seen generative models become very efficient in improving the speed and accuracy of MRI reconstruction. Existing studies have investigated the use of generative adversarial networks (GANs) [14–16]. However, GANs are difficult to train, and diffusion models have been shown to deliver better performance for medical imaging, exceeding GANs in terms of diversity and image fidelity [17]. Therefore, we concentrated on score-based models, which have the same underlying structure as diffusion-based models and allow for the mathematical integration of the MRI reconstruction process [10, 13].

In this study, we leverage the score-based generative model to accelerate breast MRI acquisition. Our approach involves training a large dataset of breast MRI images with a deep neural network to learn the underlying probability distribution of the MRI images, i.e., to learn the general appearance of these images. By combining this prior knowledge of the learned probability distribution with the acquired k-space measurements, we can quickly and accurately reconstruct MRI images and compare the image quality of reconstructions at various levels of undersampling of the k-space data.

Our hypotheses in this study were: (1) score-based models can serve as effective generative models for synthesizing breast MRI images, and (2) these models can accelerate breast MRI acquisition without compromising image quality.

## Materials and methods

### Ethics statement

Local institutional review board approval was obtained (EK028/19) and patient consent was waived for this retrospective study on anonymized data, following human and animal rights declarations and regulations.

### Patient enrolment and study design

**Fig. 1:**
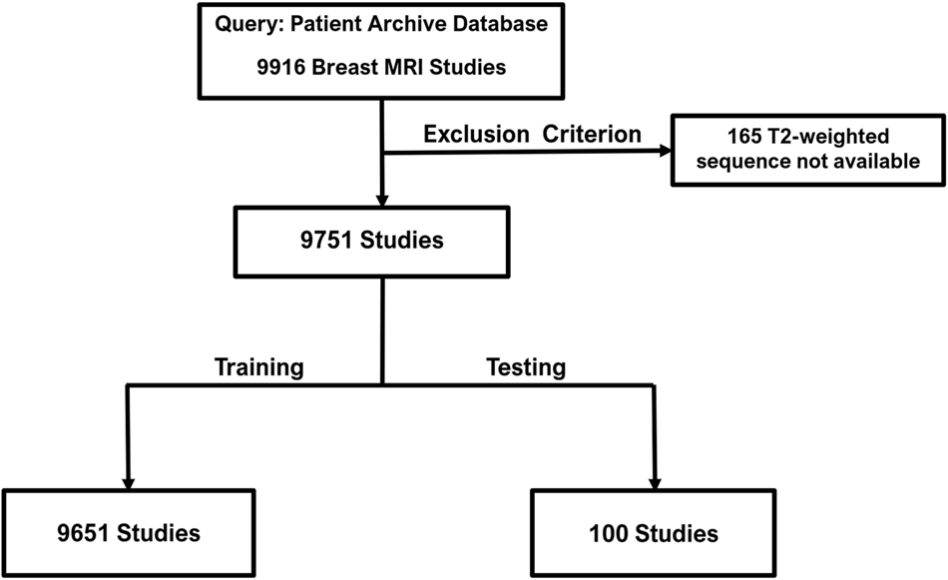
Flowchart demonstrating the data preparation process for the MR images used in this study. The dataset contained 9916 MR studies. 165 studies were discarded due to missing T2 sequences. The remaining 9751 breast MRI studies were split into two groups for training and testing

In this retrospective study, we used a dataset of breast MRI examinations from University Hospital, collected between 2010 and 2019. The dataset comprises 9751 breast MRI examinations of 5086 women. All patients were consecutively enrolled. Inclusion criteria revolved around those undergoing standard breast MRI screenings and exclusions were due to absent T2-weighted images. See data preprocessing details in Fig. 1. Dynamic Contrast Enhancement (DCE)-MRI studies of the breast were performed according to a standardized protocol on a 1.5-T system (Achieva and Ingenia; Philips Medical Systems) by using a double-breast four-element surface coil (Invivo) with two paddles being used to immobilize the breast in the craniocaudal direction (Noras) [18]. See Table S.2 in the supplemental material for a description of the T2 imaging protocol.

### Eligibility criteria

We performed the MRI reconstruction experiment using the UKA breast MRI dataset, which was constructed by randomly sampling from the set of all acquired breast MRI examinations at the hospital between 2010 and 2021. We only excluded examinations that had missing sequences, but otherwise did not use exclusion criteria to sample a comprehensive and general set of breast MRI examinations.

### MRI image reconstruction

#### Score-based model

The score-based model is a generative model that uses a U-shaped encoder-decoder network architecture [19] (Fig. S.7) to learn the probability distribution of input data via score-matching [20, 21]. For a more detailed overview of the model, refer to section A in the supplemental material.

#### Model training

We slice each of the T2-weighted 3D volumes respectively into three 2D slices whose z-position is randomly sampled in the examination. For all images, we have a total of 28,647 2D MRI slices. Image intensities were extracted and z-normalized. Image resolution was resampled to 384 × 384 for all images. We first train a prior using the score-based generative model. We used only vertical orientations during masking and other parameters remain unchanged in the algorithm. To test the reconstruction capabilities of our model, we converted the image into k-space by performing a Fourier-transform with Cartesian sampling and then deleted k-space lines at random positions according to the pre-specified ratio. E.g., for an acceleration factor of 5, we deleted 80% of all k-space lines. Comprehensive details on dataset preparation and training are available in section C of the supplemental material.

#### Image reconstruction

**Fig. 2:**
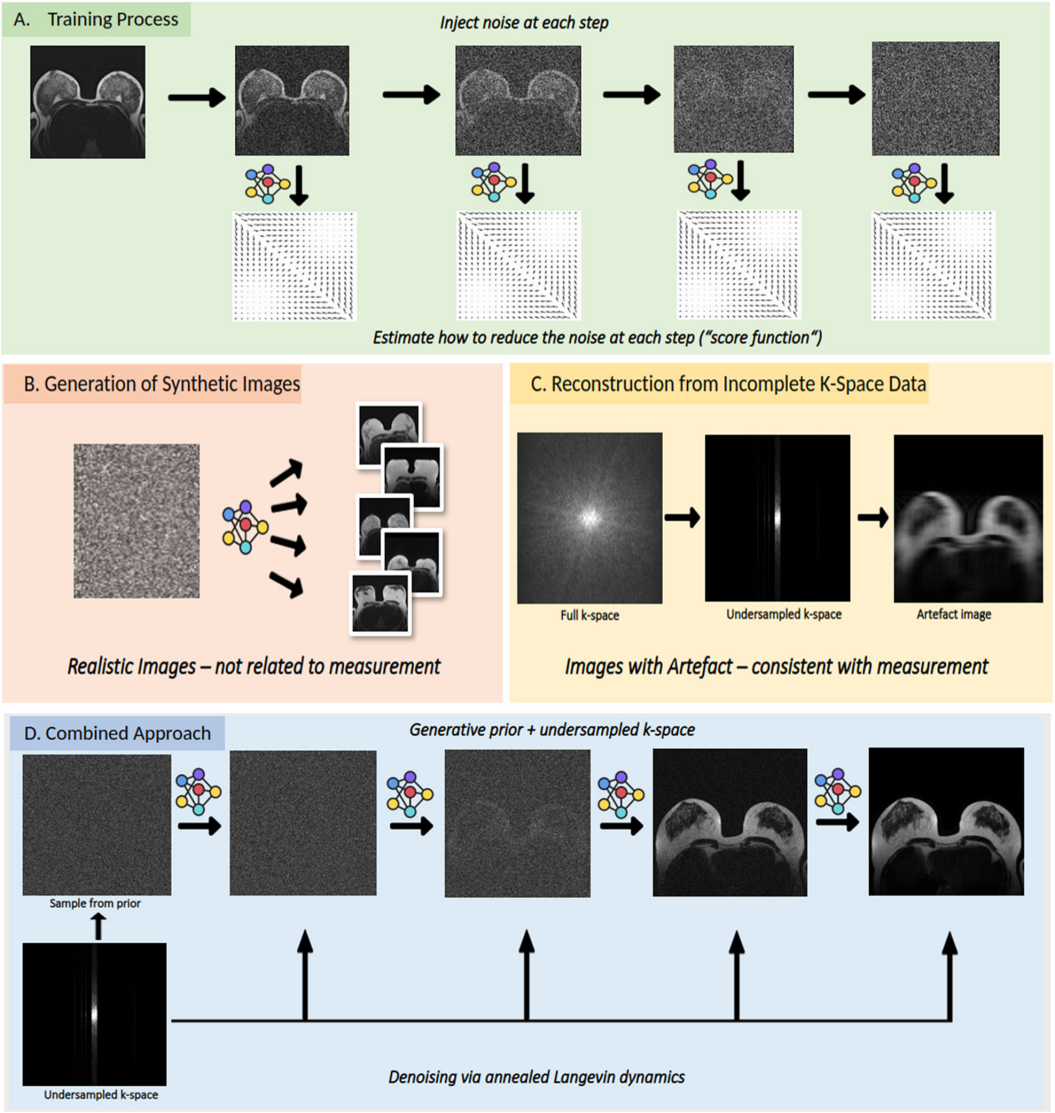
Overview of the score-based model pipeline used in this study. **A** During training, the score-based model is trained to estimate the scores of the noise-perturbed data. **B** Generation of synthetic breast MR images using the annealed Langevin to remove the noise. These images look real but does not relate to any k-space measurement. **C** Reconstruction of MRI image data from the undersampled k-space data, resulting in artifacts. **D** Combined approach which leverages the score-based model as a generative prior along with the undersampled k-space data to reconstruct high-quality breast MR images that are consistent with the measurement

With the trained model, we employed the annealed Langevin dynamics [2] for reconstructing undersampled images. To test the reconstruction capabilities of our model, we converted the image into k-space by performing a Fourier-transform with Cartesian sampling and then deleted k-space lines at random positions according to the pre-specified ratio. E.g., for an acceleration factor of 5, we deleted 80% of all k-space lines. The entire training to reconstruction procedure is illustrated in Fig. 2.

#### Image evaluation

Quantitative metrics such as peak signal-to-noise ratio (PSNR) [22] and structural similarity index measure (SSIM) [23], as well as qualitative assessments, were used. Qualitative metrics included ratings on an interval scale from two radiologists with 7 and 15 years of experience in breast MRI, respectively, comparing reconstructed images against a reference. We set up a web application for radiologists to evaluate the reconstructed MRI images on different acceleration factors and reduced k-space measurements in comparison with a reference image. As a reference image, we chose the original MRI image that had been acquired in clinical routine. They graded them on a scale of 1 to 5, with 5 symbolizing a perfect match to the reference. The evaluation focused on deviations from the reference, not just on reconstruction artifacts. A screenshot of the browser-based evaluation tool is shown in Fig. S.2 in the supplemental material.

A comprehensive statistical analysis was conducted to evaluate the performance of the image reconstruction method and to assess the agreement between the evaluating radiologists.

##### Quantitative evaluation

Quantitative similarity between the reconstructed and fully sampled images was assessed using metrics like PSNR and SSIM. A sample of 100 slices from different examinations was utilized to compute average PSNR and SSIM values. Trends in these metrics with varying undersampling factors were analyzed, revealing implications for data compression and resulting image quality.

##### Qualitative evaluation

Radiologist ratings, conducted on a five-point scale, were averaged and standard deviations were calculated for various acceleration factors. Inter-reader agreement between the two radiologists was evaluated using Cohen’s kappa statistic.

##### Statistical significance

A non-parametric Mann–Whitney *U* test was performed to determine the significance in ratings. Results were interpreted considering a confidence level of 95%.

##### Reading strategy

To ensure a standardized and unbiased assessment, radiologists were informed about the study objectives and given a reference set. Their qualitative assessments were further supported by a screenshot of the browser-based evaluation tool, as presented in Fig. S.2 in the supplemental material.

##### Statistical significance

The significance of the differences in ratings was determined using the non-parametric Mann–Whitney *U* test, interpreted at a 95% confidence level.

## Results

### Dataset and demographics

Our final cohort, after applying the inclusion and exclusion criteria, consisted of 9751 examinations from 5086 patients. The demographic details of the patients are summarized in Table S.1. The examinations, which spanned from January 2010 to December 2019, show that 37% were acquired in a screening setting, 37% were follow-up examinations, and the remaining 26% comprised examinations performed for problem-solving and other reasons (details can be found in Fig. S.1).

### Image reconstruction

**Fig. 3:**
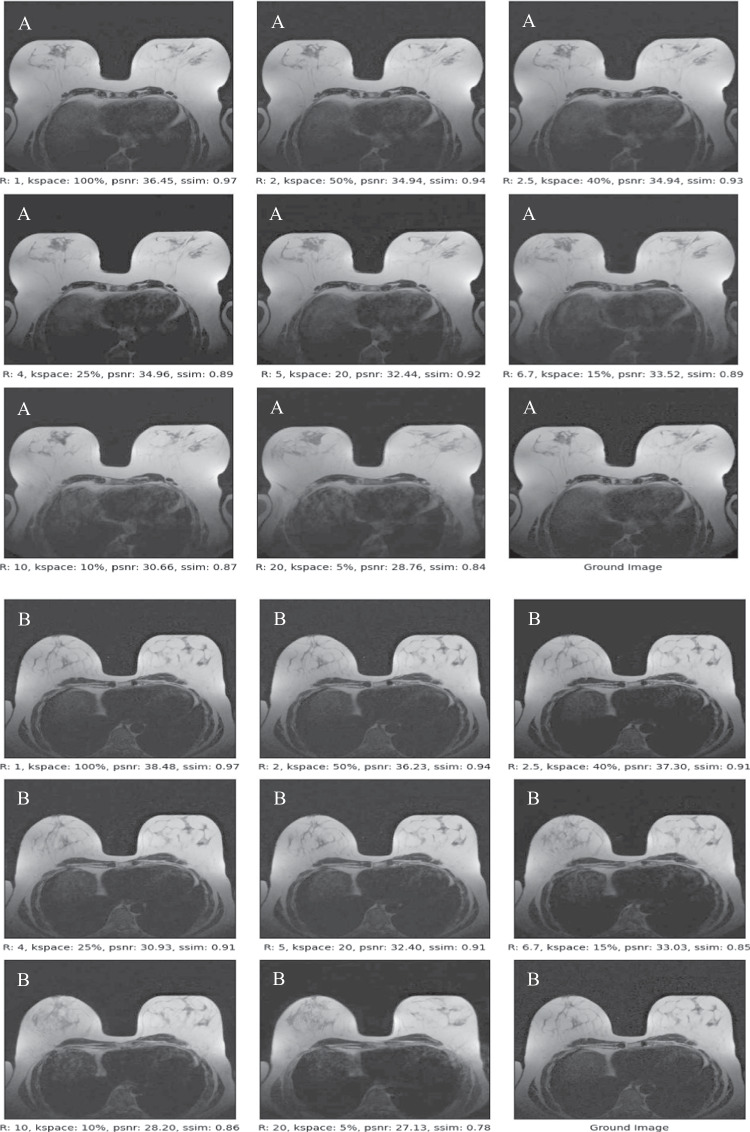
Two reconstructed breast MRI slices (**A**, **B**) at different acceleration factors for two illustrative examples. The fully sampled ground truth image is shown in the right lower corner of each subfigure. Score-based reconstructed images are shown in the remaining eight panels of each subfigure with the acceleration factor R increasing from R = 1 (full sampling) to R = 20 (twentyfold undersampling). Quantitative PSNR and SSIM scores are shown below each figure. Note that the images look realistic, but exhibit anatomical variations as compared to the ground truth images

First, to test the trained model generative capabilities, we were able to sample random breast MRI images without any k-space data (see Fig. S.3 in the supplemental material). Second, the model effectively reconstructed images from undersampled k-space data. Figure 3 displays MRI slices from two examinations based on different sampling rates.

### Quantitative evaluation

The similarity between the reconstructed and the fully sampled images was measured using PSNR and SSIM. We computed the average PSNR and SSIM values across 100 randomly chosen slices from 100 different exams. As evident from Fig. S.4 in the supplemental material, the average PSNR and SSIM values showed a decline with higher undersampling factors. At the maximum undersampling factor of R = 20, the score-based method still showed a PSNR of approximately 27.04 dB and an SSIM index of 0.78.

### Qualitative evaluation

**Fig. 4:**
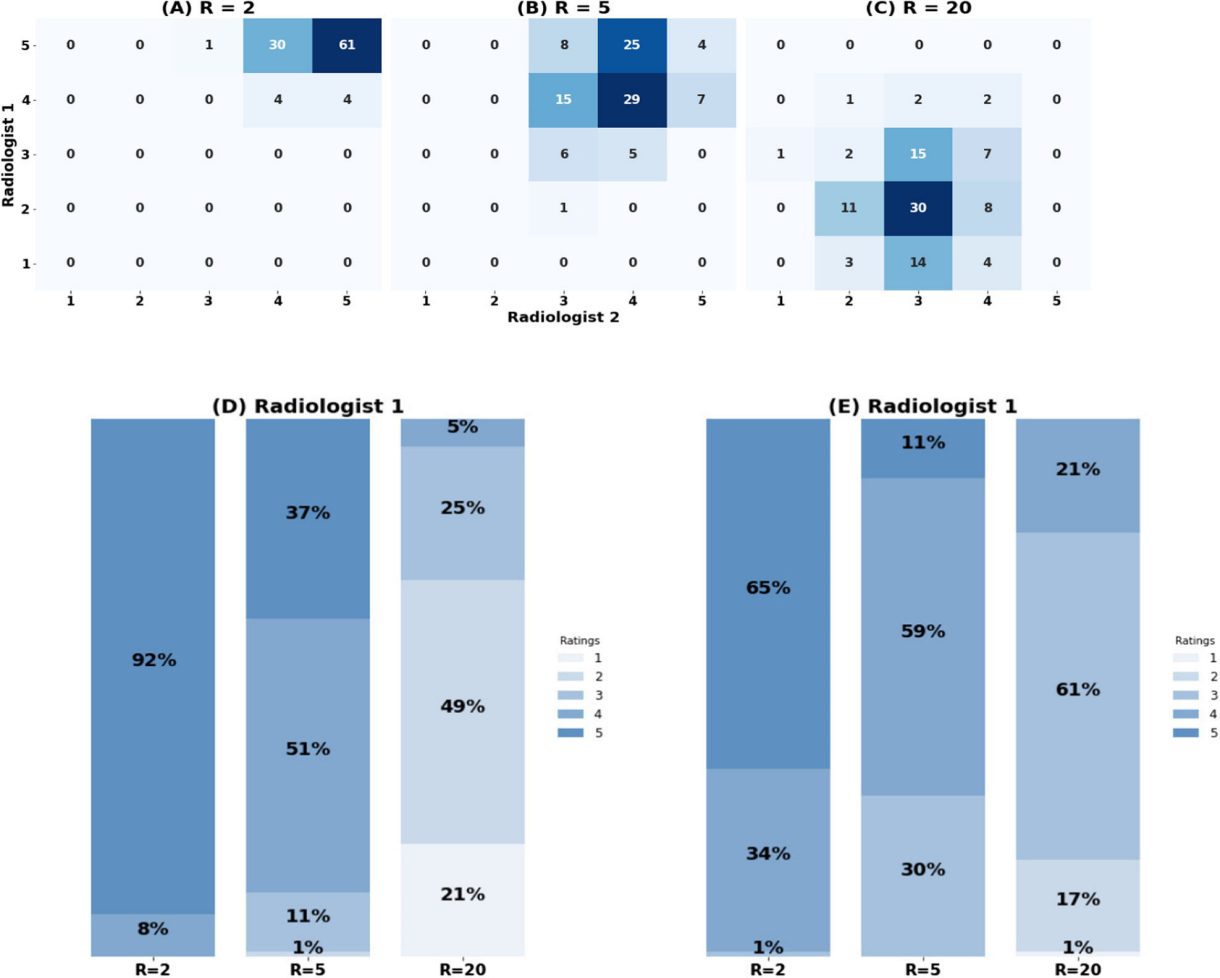
Rating for the images that were reconstructed from the undersampled k-space data. **A**–**C** Show the confusion matrix for the radiologists’ ratings for acceleration factors of R = 2, 5, and 20 respectively. **D** Rating allocated by the radiologist with 7 years of experience for R = 2, 5, and 20 respectively. **E** Rating allocated by the radiologist with 15 years of experience for R = 2, 5, and 20 respectively

Two radiologists rated the reconstructed images using a scale from 1 to 5. The mean ratings for acceleration factors R = 2, R = 5, and R = 20, were 4.8 ± 0.10, 4.0 ± 0.10, and 2.6 ± 0.10 respectively (see Fig. 4). A significant inter-reader agreement was observed with a Cohen’s kappa value of 0.61 (95% confidence interval: 0.56–0.68). The Mann–Whitney *U* test revealed a *p*-value of 0.26. Figure S.6 in supplemental material provides instances of reconstructed images that exhibited notable rating differences among the radiologists.

### Correlation between quantitative metrics and qualitative ratings

To bridge the gap between quantitative image quality metrics and qualitative expert evaluations, we conducted an analysis that directly compares the PSNR and SSIM values with the subjective ratings provided by two anonymous radiologists. This comparison, illustrated in Fig. S.5, serves to examine the extent to which objective measures of image reconstruction quality align with professional clinical judgment.

The analysis presented in Fig. S.5 through box plots shows the distribution of PSNR and SSIM values across various rating categories established by the radiologists. The plots reveal a positive correlation, indicating that higher PSNR and SSIM values generally correspond to higher qualitative ratings. This relationship validates the predictive value of PSNR and SSIM as indicators of image quality from a radiological perspective. Specifically, the central tendency of the metrics across ratings highlights a trend where improved quantitative measures—indicative of closer approximations to the fully sampled image—are associated with more favorable assessments by the radiologists.

### Computational details

The score-based model’s training was executed on 28,647 images, taking around 72 h on an NVIDIA RTX 3090 GPU. In practice, the pre-trained model would be used for image reconstruction, taking about 7 min per MRI slice on the same GPU.

## Discussion

Our study highlighted the potential of the score-based diffusion model to streamline MRI reconstruction, presenting an acceleration in MRI while maintaining clarity and reliability. As the acceleration factor increased, we observed that both quantitative measures (PSNR and SSIM) and qualitative evaluations by radiologists showed a decrease in image quality. Notably, the model’s reconstructions from undersampled k-space data reached acceleration factors as high as R = 20, though image quality ratings reduced considerably for this highest acceleration level.

In addition to our findings, it is important to acknowledge that while PSNR and SSIM provide valuable insights into the technical quality of image reconstruction, they may not fully encapsulate the complexities of visual assessments conducted by radiologists. The presence of outliers in our data suggests that evaluations by radiologists are influenced by a range of factors that extend beyond the simple metrics of image fidelity. Factors such as the clinical relevance of the images, the radiologists’ years of experience, and the perceptibility of important diagnostic features play significant roles in their judgments. This observation underlines the notion that quantitative metrics, while helpful, do not fully capture the complexities of visual assessment and clinical applicability as perceived by medical experts [24]. Our findings generally showed good agreement between the quantitative evaluations of our reconstruction technique and the qualitative assessments by radiologists; however, this highlights the need for a cautious interpretation of these metrics in clinical settings. Future research should consider these dynamics to better understand how such tools can be integrated into clinical practice without over-reliance on quantitative metrics alone.

Presently, acceleration techniques such as sensitivity encoding (SENSE) [3] and generalized autocalibrating partially parallel acquisitions (GRAPPA) [25] dominate clinical practice, with emerging research focusing on convolutional neural networks for MRI reconstruction [26, 27]. Yet, our study emphasizes the unique advantages of the score-based model. Primarily, it sidesteps the need for multiple coils, a requisite for SENSE and GRAPPA. Our results evidenced this with an achieved acceleration factor of 20, even when using just four coils. Further, the versatility of the score-based model allows it to adapt to various acquisition schemes post-training, a contrast to specialized neural networks which demand specific retraining for different acquisition techniques [26, 27].

Furthermore, we incorporated both T1 and T2 weighted MRI sequences as class labels into the score diffusion model. This approach allowed us to train a single model that could handle multiple MRI sequences rather than separate models for each sequence. In preliminary experiments, we also tested whether a model that was only trained on T2-weighted images could be used to reconstruct T1-weighted images, but we found that we needed to integrate T1-weighted images for this to work. This integration is beneficial as it reduces the computational cost of training separate models and improves the accuracy and efficiency of MRI reconstruction.

However, to minimize variability, it is crucial for radiologists to adhere to established guidelines and protocols for image interpretation. Consulting with colleagues or referring to other imaging modalities may also be necessary. A significant limitation of our study is that the diagnostic accuracy of the reconstructed images has not been evaluated. For instance, it is conceivable that reconstruction methods based on generative AI models might not accurately represent potentially malignant lesions. While no evidence of this issue was found, the lack of evidence does not confirm the absence of a problem. Thus, extensive investigations are needed before these methods can be reliably used in clinical settings. Should future studies show that the presented methods can be implemented in clinical practice, then that would help alleviate the problem of limited MRI capabilities available to patients. This is particularly pressing considering the recent recommendation of the European Society for Breast Imaging to screen women with dense breasts by means of MRI [28].

Another approach to employing generative models for breast imaging with MRI is to reduce the need for contrast agents. As demonstrated in a recent publication by Müller-Franzes et al [7], generative models can enhance MRI subtraction images with reduced contrast doses. However, we did not train a model for different anatomical regions or employ the model on external institutions. As a proof-of-concept study, we intended to show that the generative model learns the underlying distribution of breast images at the institution where it had been trained. There is evidence, however, that a model trained on one specific anatomy or dataset can also be used for the reconstruction of unrelated anatomies: Jalal et al trained a score-based diffusion model to reconstruct MRI images of the brain and applied it to abdominal and knee MRI images [13]. Future research should investigate this and perform clinical evaluations of such reconstructed images.

Despite the promising outcomes, our study isn’t devoid of limitations. First, our score-based model’s dependency on a vast set of fully sampled images for training could limit its adaptability across diverse datasets or higher-resolution images. This raises pertinent questions about its generalization capacities which necessitate further exploration. Moreover, our study’s scope was limited to two-dimensional imaging, and understanding its efficacy on three-dimensional imaging remains a topic for future investigations. Our focus was also predominantly on T2-weighted images; thus, extrapolating our findings to varied MRI contrasts requires additional research. Last, the inherent nature of generative models to possibly “hallucinate” information is a crucial consideration, especially when assessing their viability in clinical settings. The reliability of such models in clinical scenarios remains an essential avenue for future studies.

In conclusion, our work demonstrates the potential of score-based models for the acceleration of MRI reconstruction. In contrast to existing approaches, the score-based model does not require multiple coils and can be used with arbitrary acquisition schemes. Further research is needed, but we reckon that score-based models are a promising approach for accelerating MRI reconstruction in clinical practice.

## Supplementary information

ELECTRONIC SUPPLEMENTARY MATERIAL

## Abbreviations

GRAPPA: Generalized autocalibrating partially parallel acquisitions
MRI: Magnetic resonance imaging
PSNR: Peak signal-to-noise ratio
SENSE: Sensitivity encoding
SSIM: Structural similarity index measure
