# Supplementary material for: Accelerating breast MRI acquisition with generative AI models
Source: Eur Radiol. 2024 Aug 1;35(2):1092–100. doi: 10.1007/s00330-024-10853-x (PMC11782449; doi:10.1007/s00330-024-10853-x)
Supplement: Supplementary file 1 — ELECTRONIC SUPPLEMENTARY MATERIAL [file 330_2024_10853_MOESM1_ESM.pdf]

Accelerating Breast MRI Acquisition with Generative AI Models

ELECTRONIC SUPPLEMENTARY MATERIAL

A. Inverse Problem and Score-based Model

The inverse problem refers to the mathematical process of reconstructing an image of the internal structure of the body from the signals received by the MRI machine. Let $y \in R^m$ be a measured signal of a vector of m linear measurements such that

$$y = Ax + \varepsilon, \qquad \text{(S.1)}$$

where $A \in R^{mxn}$ is a linear forward operator of Fourier encoding matrix and $\varepsilon \in R^n \sim N(0, \sigma^2 I)$ is the acquisition white Gaussian noise vector and $x \in R^n$ is the medical image of n pixels. We have

$$y = Ax + \varepsilon \sim p(x)$$

such that the conditional posterior distribution

$$p(x \mid y) = \frac{p(x)p(y \mid x)}{p(y)} \qquad \text{(S.2)}$$

is determined by the gradient of the log probability of the Bayes' formulation to yield the conditional score

$$\nabla_x lo\, g\, p(x \mid y) = \underbrace{\nabla_x log\ p(x)}_{Score\ function} + \underbrace{\nabla_x log\ p(y \mid x)}_{Acquisition\ process}, \qquad \text{(S.3)}$$

where $p(x)$ is the true data distribution. By adding multiple noise levels to $p(x)$, we can obtain an *i*[th] sequence of noise perturbed distributions $q_{\sigma_i}(x_i) = \int p(x_i \mid x_{i-1})p(x_{i-1})dx_{i-1}$ that converge to the true data distribution $p(x)$. The conditional perturbation distribution according to the step *i* is

$$q_{\sigma_i}(\bar{x}|x) = \mathcal{N}(\bar{x} \mid x, \sigma_i^2 I).$$

That is, $\bar{x} = x + \sigma_i z$, where $z \sim \mathcal{N}(0, I)$ and $\frac{\sigma_1}{\sigma_2} = \frac{\sigma_2}{\sigma_3} = \cdots = \frac{\sigma_{L-1}}{\sigma_L} > 1$ a sequence of noise scales for $i = 1, \ldots, L$. We now define $s_\theta(\bar{x}, \sigma_i) \approx \nabla_x log\ q_{\sigma_i}(x)$ a score model that depend on sigmas. The conditional score $s_\theta(\bar{x}, y, \sigma_i)$ is estimated by first training the unconditional score function $s_\theta(\bar{x}, \sigma_i)$ on the objective function.

$$L(\theta, \{\sigma_i\}_{i=1}^{L}) = \frac{1}{L}\sum_{i=1}^{L}\lambda(\sigma_i) l(\theta; \sigma_i),$$

where $l(\theta; \sigma_i) = \frac{1}{2}\mathbb{E}_{p(x)}\mathbb{E}_{\bar{x}\sim\mathcal{N}(x, \sigma_i^2 I)}\left[\|s_\theta(\bar{x}, \sigma_i) + \frac{\bar{x}-x}{\sigma_i^2}\|_2^2\right]$ and then combined with the auxiliary model $\nabla_x log\ p(y \mid x)$ which represents the process of acquisition typically in a frequency domain called k-space. Given the random variables x and y, the general inverse problem aims to generate samples from the conditional posterior distribution $p(x \mid y)$. We can estimate the score of the learned prior distribution $p(x)$ from the trained score model and estimate the conditional score of y from x from the acquisition process $p(y \mid x)$. However, there are different approximation techniques for solving this problem.

B. Posterior sampling Method

We employed the image reconstruction algorithm developed by Jalal et al. [9]. Our aim is to reconstruct the image x which represents the pixel values of the image, from an undersampled measurement y, which represents the k-space measurement. The algorithm approximates the image using a stepwise approach by iteratively refining the estimate of x. When we have a given undersampled measurement y, there is a set of potential images that agree with the measurement, which is described by the probability distribution $p(x \mid y)$. This means that $p(x \mid y)$ is the distribution of images x that agree with the measurement y.

We cannot directly access this distribution, but we can calculate the k-space measurement y for a specific image x by performing a Fourier transform and deleting k-space lines that have not been measured. This is described by equation (S.1). By using Bayes' rule, equation (S.2) relates these two quantities and enables the calculation of the derivative of $p(x \mid y)$. To relate $p(x \mid y)$ with $p(y \mid x)$, we need to know the distribution of images x per se, which is the prior distribution of images without the knowledge of any measurement. This is provided by the score-based model via the score function in equation (S.3). It is important to note that we cannot directly calculate $p(x \mid y)$, but only its derivative, which is why a stepwise approach is required: we update the image x starting from pure noise by adding the stepwise derivatives of $p(y \mid x)$ and the prior $p(x)$, thereby ultimately

arriving at the maximum of $p(x \mid y)$, i.e. the highest probability where image x corresponds to the undersampled measurement y.

In more detail, this process is achieved using the Langevin Dynamics algorithm. We briefly highlight the Langevin Dynamics algorithm for sampling from the posterior distribution below. Given the observation y, the posterior sampling recovery algorithm outputs some $\bar{x}$ according to the posterior distribution $p(x \mid y)$. The Langevin Dynamics is used to sample from the posterior $p(x \mid y)$ by running noisy gradient ascent

$$x_{t+1} \leftarrow x_t + \eta_t \nabla_{x_t} log\ p(x_t \mid y) + \sqrt{2\eta_t}\zeta_t, \qquad \zeta_t \sim \mathcal{N}(0,1).$$

As the number of iterations $t \to \infty$ and step size $\eta_t \to 0$, the Langevin dynamics will correctly sample from the converged posterior $p(x \mid y)$. The authors proposed the annealed Langevin Dynamics, where the marginal distribution of x_t is modelled as $p_t = p * \mathcal{N}(0, \beta_t^2)$ and trained to estimate the score function

$$f(x_t; \beta_t) := \nabla_{x_t} \log \left((p * \mathcal{N}(0, \beta_t^2)(x_t) \right),$$

where $\beta_1 < \beta_2 < \cdots < \beta_T$ and $\{\beta_t\}_{t=0}^{T-1}$ represent the increasing standard deviations over a total of T time steps. They proposed an auxiliary model in the form $\nabla_{x_t} log\ p(y \mid x_t) = \frac{A^H(y - Ax_t)}{\sigma^2 + \gamma_t^2}$ where A^H is the Hermitian transpose of A and $\gamma_t \to 0$ is a decreasing sequence, thus

$$\nabla_{x_t} log\ p(x_t \mid y) \approx f(x_t; \beta_t) + \frac{A^H(y - Ax_t)}{\sigma^2 + \gamma_t^2}$$

And the final algorithm yields: for $x_0 \sim U(0,1)$ and for all $t = 0, \cdots, T-1$,

$$x_{t+1} \leftarrow x_t + \eta_t \nabla_{x_t} lo\, g\, p(x_t \mid \mathrm{y}) + \sqrt{2\eta_t}\zeta_t, \qquad \zeta_t \sim \mathcal{N}(0, I)$$

Algorithm 1: Reconstruction Algorithm

1. **Initialize** $\mathbf{x_0} \sim \boldsymbol{\mathcal{U}}(\mathbf{0}, \mathbf{1})$
2. **for** $\boldsymbol{t} = \mathbf{0}, \cdots, \boldsymbol{T} - \mathbf{1}$ **do**
3. $\quad \boldsymbol{\zeta_t} \sim \boldsymbol{\mathcal{N}}(\mathbf{0}, \boldsymbol{I})$
4. $\quad \nabla_{\mathbf{x}_t} \mathbf{log}\ \boldsymbol{p}(\mathbf{x}_t \mid \mathbf{y}) \approx \boldsymbol{f}(\mathbf{x}_t; \boldsymbol{\beta}_t) + \dfrac{\mathbf{A}^{\boldsymbol{H}}(\mathbf{y} - \mathbf{A}\mathbf{x}_t)}{\boldsymbol{\sigma}^2 + \boldsymbol{\gamma}_t^2}$
5. $\quad \mathbf{x}_{t+1} \leftarrow \mathbf{x}_t + \boldsymbol{\eta}_t \nabla_{\mathbf{x}_t} \mathbf{log}\ \boldsymbol{p}(\mathbf{x}_t \mid \mathbf{y}) + \sqrt{\mathbf{2}\boldsymbol{\eta}_t}\boldsymbol{\zeta}_t$
6. **end for**

C. Experimental Results

C.1. Dataset

We performed the MRI reconstruction experiment using the University Hospital breast MRI dataset. The indications for the MRI scans are given in Figure S.1 with the majority of studies (37%) being performed for screening for breast cancer and another 37% as follow-up examination. All patient information was anonymized during data preprocessing. The forward operator A^H which is a Cartesian acquisition comprises of a two-dimensional discrete Fourier Transform $\hat{A}$ and an undersampling mask M . The Fourier transform is first applied to the medical image x and the resulting image is then undersampled with the undersampling mask to select the number of sampled lines from the k-space data. Fully sampled lines through the centre of the k-space matrix constitute the autocalibration signal (ACS) region. This ACS region is computed based on the acceleration factor and sample size. We keep only 50% of the sampled lines for the ACS lines and ensure to keep this proportion for all values of the acceleration factor.

C.2. Training and Reconstruction

A total of 9,751 examined cases were used for training. Each of the T2-weighted 3D volumes was sliced into three 2D slices for training purposes. In total, we obtained 29,253 2D MRI slices from all images. Initially, we employed a U-Net architecture (see Figure S.6), widely recognized for its efficiency in medical image segmentation tasks, to train our model via score matching. We then trained prior using the score-based generative model on the 28,647-slice training set. The Noise Conditional Score Networks version 2 (NCSNv2) model architecture, along with the Python codes used for training the score-based model, can be found in the GitHub repo: https://github.com/ermongroup/ncsnv2. Subsequently, we reconstructed a single slice from each of the 100 test MRI datasets using the posterior sampling algorithm (See Algorithm 1). We trained the images at a resolution of 384 × 384 on 2 channels, each channel representing the real and imaginary parts of the k-space measurements. The 2-channeled image is retained during the reconstruction process to be consistent with the dimensions of the k-space data and the model architecture. After reconstruction, we convert the image back to 1 channel by taking the magnitude of the complex pixel values to obtain the real-valued pixels of the image. The MRI images are reconstructed at different acceleration values (R) representing the ratio of the full k-space lines to the total sampled lines.

As shown in Figure 3, the method was able to reconstruct breast MRI images at different acceleration factors. Windowing was applied to the reconstructed T2-weighted images to map intensity values to brightness values. Quantitatively, the PSNR and SSIM average scores (see Figure S.4) were used as metrics to evaluate the reconstructed images compared to the ground truth image. Qualitatively, the reconstructed images were also evaluated using the ratings from 2 experienced radiologists.

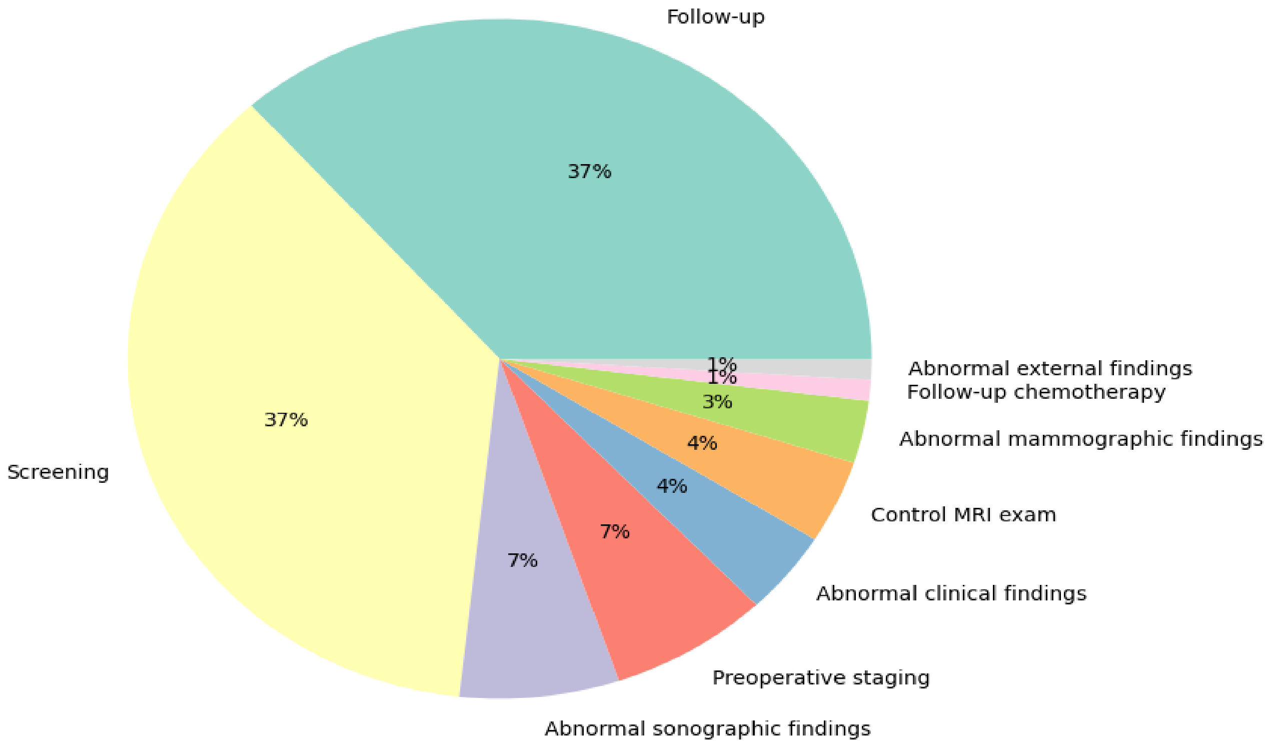

Figure S.1: Indications for the MRI examinations. In total, 9,751 examinations were employed in this study. 37% of all examination were acquired in the screening setting, another 37% were follow-up examinations, the remaining 26% contained examinations performed for problem-solving and other reasons.

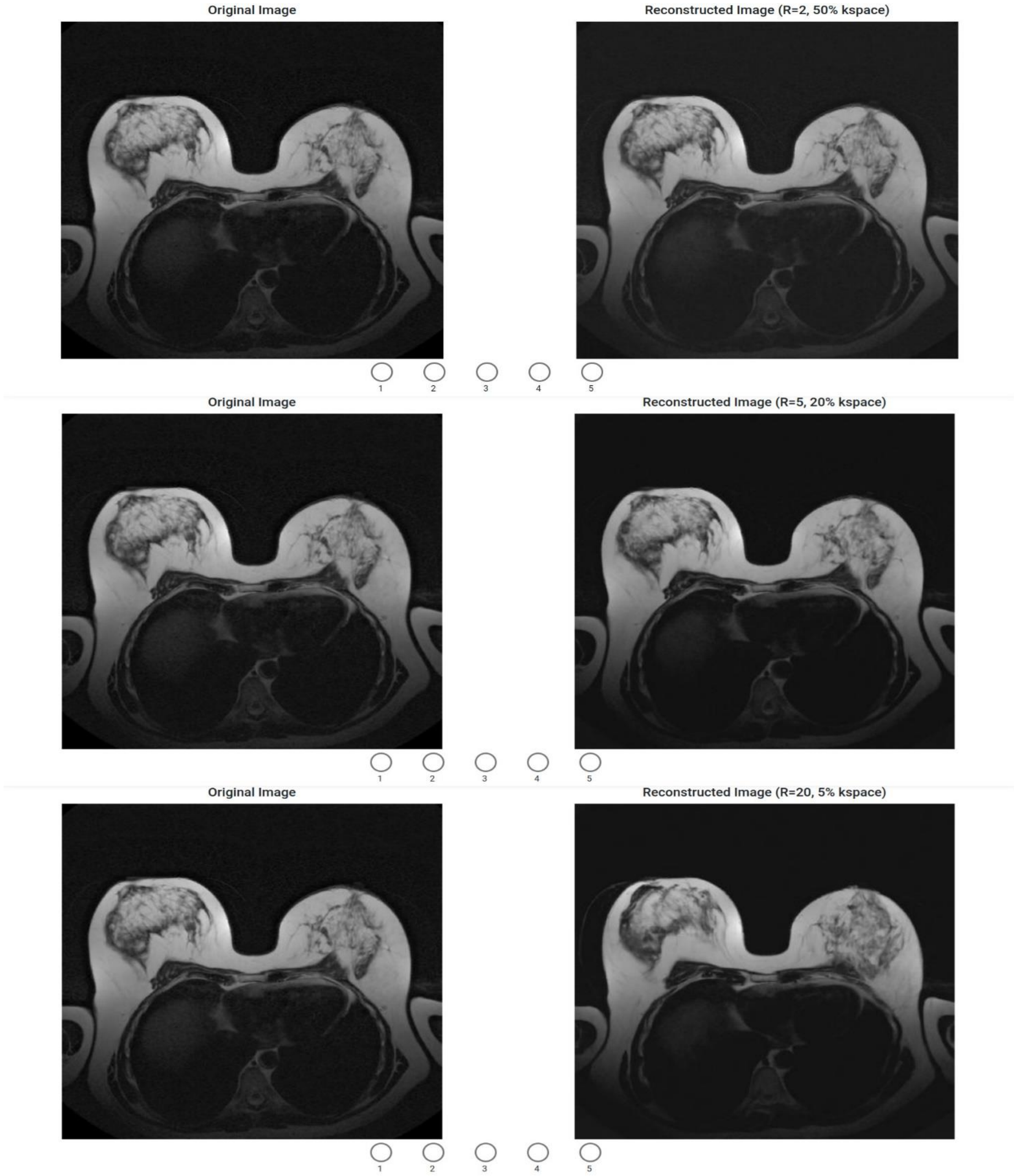

Figure S.2: Figure showing the browser-based setup used for clinical evaluation of the reconstructed breast MRIs. Each row displays the original image on the left and the reconstructed image on the right at different acceleration values, R=2, R=5, R=20. The radiologists were asked to rate the reconstructed images on a scale of 1 to 5 using the input button provided.

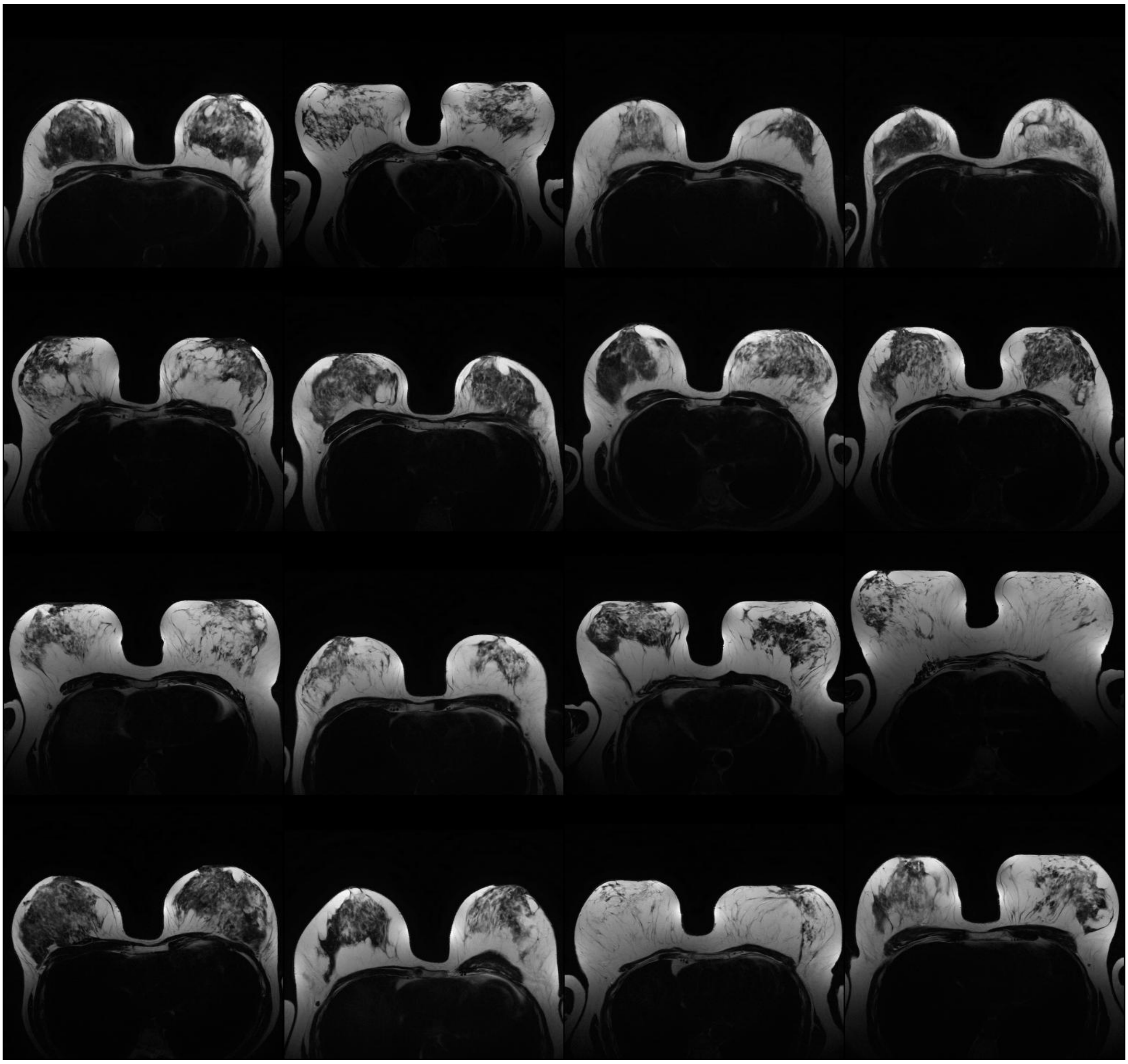

Figure S.3: Synthetic Breast MRI Slices: These images were generated from pure noise without any k-space data by the trained score-based model, showcasing the model's ability to produce high-quality synthetic breast MRI images.

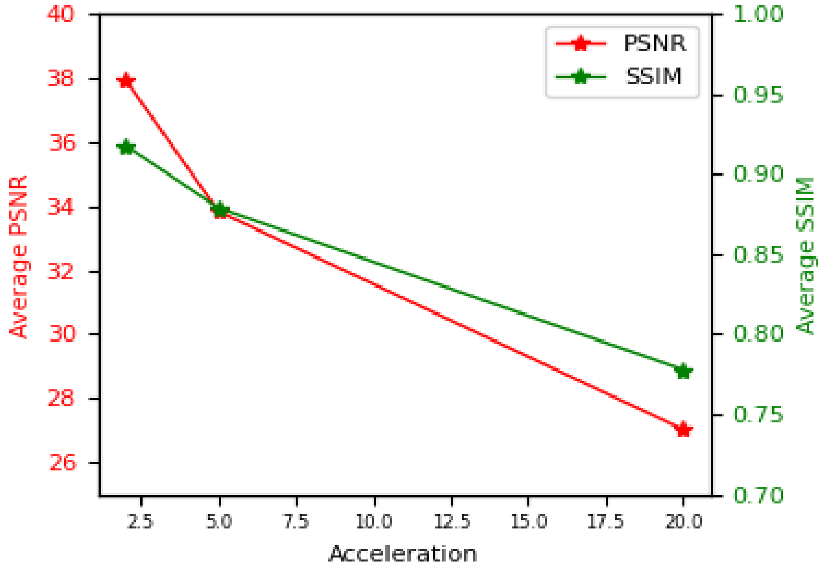

Figure S.4: This figure displays the average performance of the score-based method across 100 MRI reconstructed slices in terms of PSNR and SSIM metrics at different acceleration factors. For an acceleration factor of 2, the mean SSIM is 0.91 (95% confidence interval: 0.91 - 0.93), and the mean PSNR is 38.19 dB (95% confidence interval: 37.66 dB - 38.73 dB). At an acceleration factor of 5, the mean SSIM is 0.88 (95% confidence interval: 0.87 - 0.89) and the mean PSNR is 34.25 dB (95% confidence interval: 33.77 dB - 34.73 dB). For an acceleration factor of 20, the mean SSIM is 0.78 (95% confidence interval: 0.77 - 0.80) and the mean PSNR is 27.30 dB (95% confidence interval: 26.87 dB - 27.72 dB).

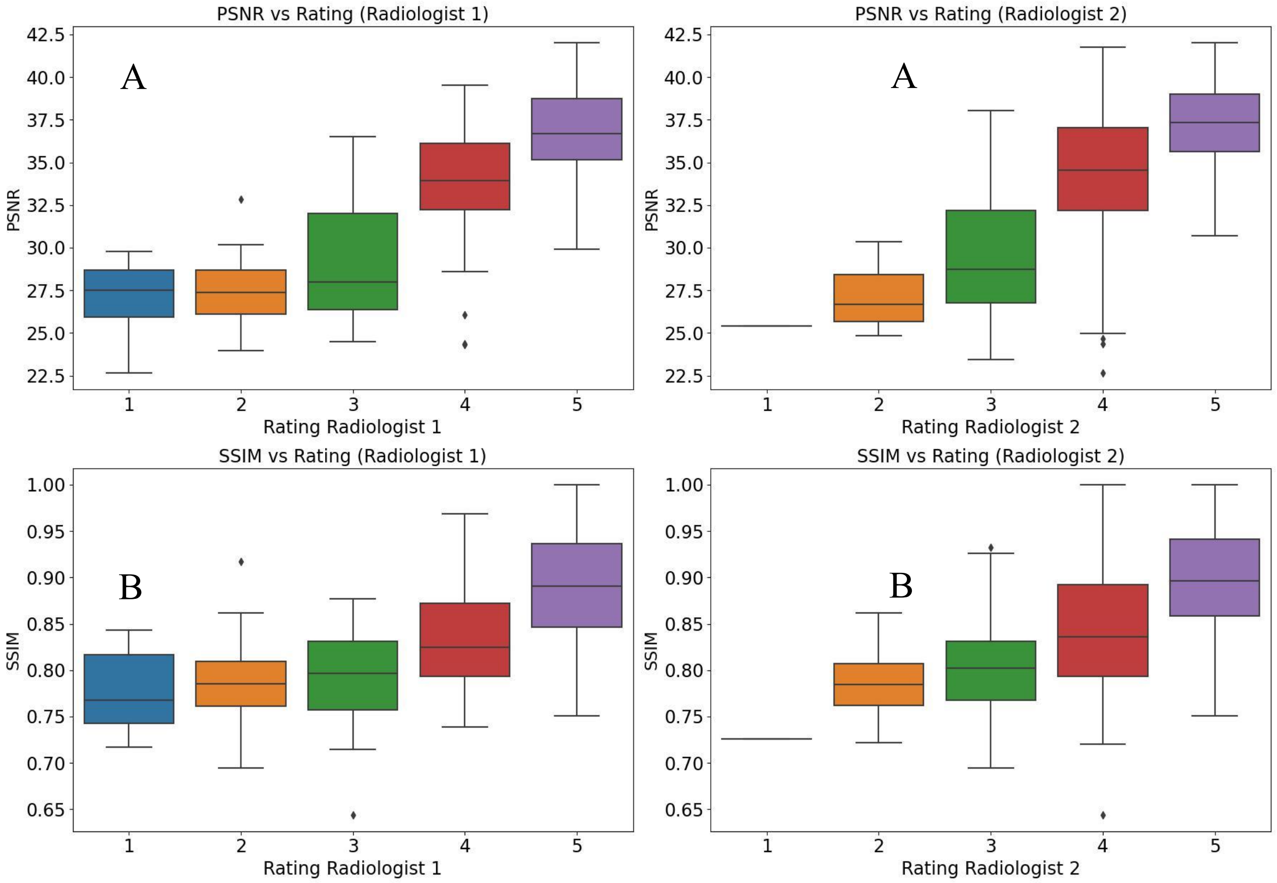

Figure S.5: The box plots of PSNR (A) and SSIM (B) metrics plotted against the ratings provided by two anonymous radiologists, illustrating a positive correlation between quantitative image quality metrics and expert qualitative evaluations. The box plots reveal the central tendency, dispersion, and outliers in the distribution of PSNR and SSIM values across different rating categories, revealing a general trend where higher ratings are associated with higher PSNR and SSIM values.

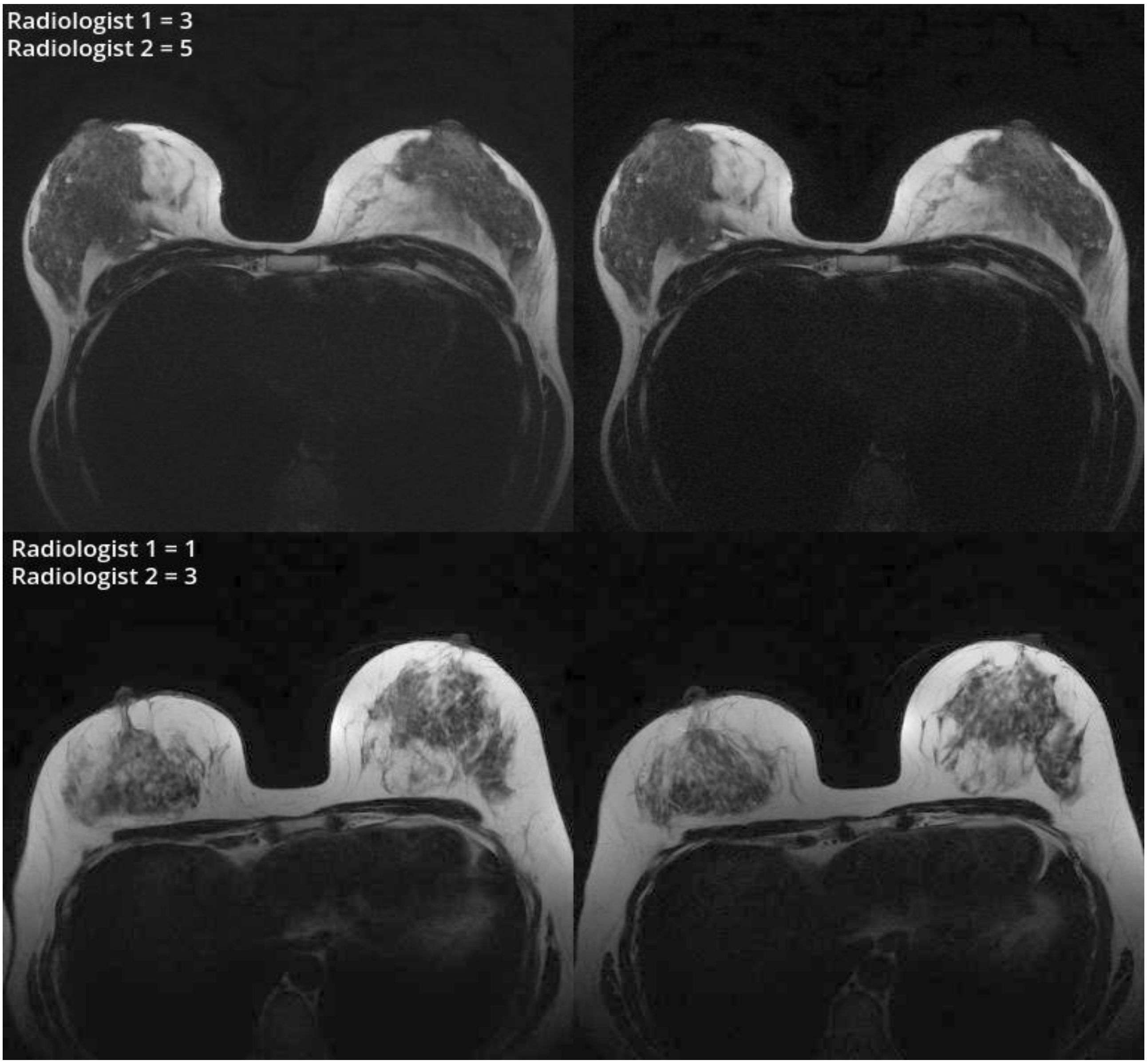

Figure S.6: The examples illustrated here represent two examination cases, one with R=2 (top row) and another with R=20 (bottom row), where the ratings given by the two radiologists were notably divergent. The first image on the left in each row is a reconstruction from the undersampled k-space data, while the image on the right is the actual image. The ratings provided by the two radiologists are displayed at the top left corner of each reconstructed image.

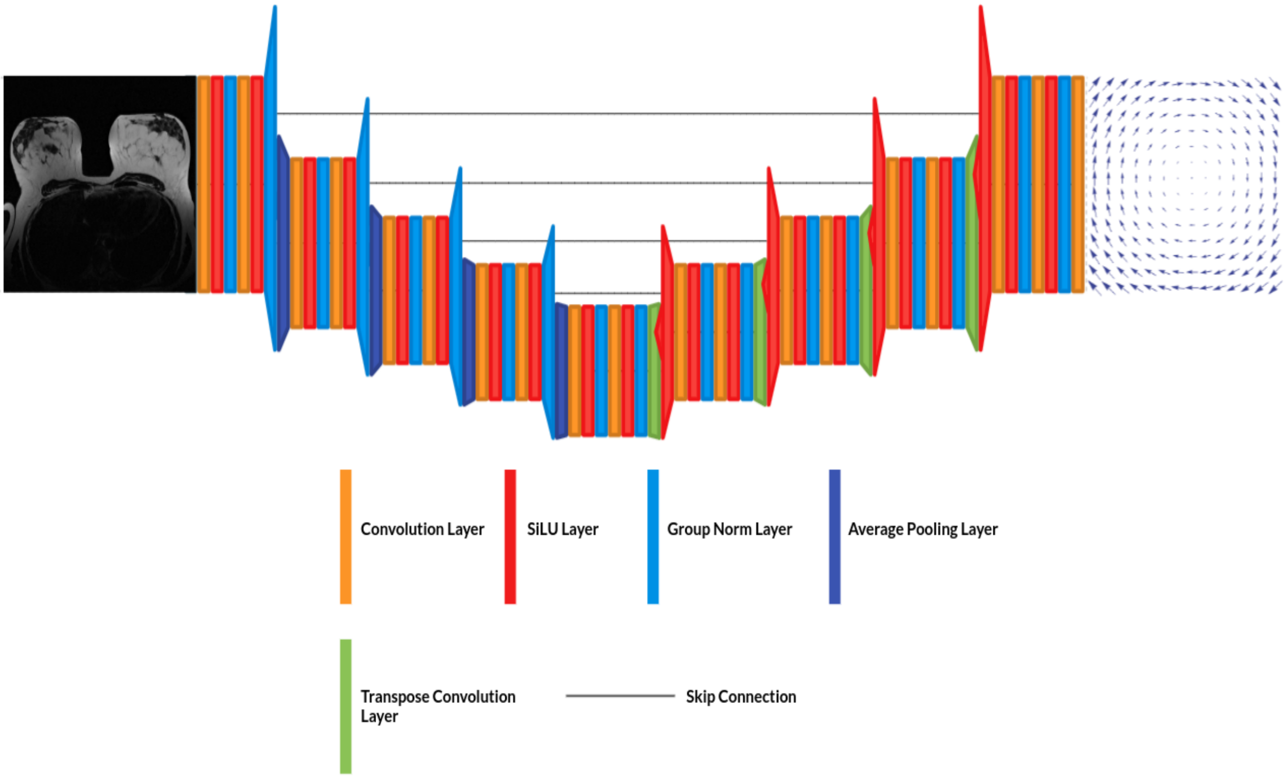

Figure S.7: Diagram of the U-Net architecture for the score-based model consisting of an encoder and a decoder network with skip connections. The encoder consists of a series of convolution layers with an increasing number of filters and decreasing spatial dimensions, followed by group normalization (GroupNorm) and Sigmoid Linear Units (SiLU) non-linear activation. The decoder consists of a series of transposed convolution layers with a decreasing number of filters and increasing spatial dimensions, also followed by a GroupNorm and SiLU non-linear activation function. The skip connections connect the corresponding layers between the encoder and decoder networks, allowing the model to use high-level and low-level features for better score estimation.

Characteristic	Full Data Set	Training Data Set	Test Data Set
No. of Patients	5086	4986	100
Patient age (mean ± SD, range)	55 ± 11 (17-91)	55 ± 11 (16 - 91)	56 ± 11 (35-79)
No. of MRI examinations	9751	9579	100

Table S.1: Description of the Breast-MRI dataset

Orientation	Axial	
Field Strength [T]	1.5	
	DCE	T2
Acquisition Type	2D	2D
Echo Type	Gradient Echo	Turbo Spin Echo
Fat Suppression	No	No
TR [ms]	264 ± 22	4008 ± 201
TE [ms]	4.6 ± 0.10	110
Flip angle [°]	90 ± 30	90
Slice Thickness [mm]	3.1 ± 0.20	3.1 ± 0.20
Number of slices	28 ± 2	33 ± 2
Matrix X	520 ± 22	535 ± 39
Matrix Y	520 ± 22	535 ± 39
Field of View X [mm]	333 ± 26	332 ± 26
Field of View Y [mm]	333 ± 26	332 ± 26

Table S.2: Acquisition Parameters for Breast MRI
